# Reduced respiratory motion artefact in constant TR multi-slice MRI of the mouse

**DOI:** 10.1016/j.mri.2019.03.018

**Published:** 2019-07

**Authors:** Paul Kinchesh, Philip D. Allen, Stuart Gilchrist, Veerle Kersemans, Simone Lanfredini, Asmita Thapa, Eric O'Neill, Sean C. Smart

**Affiliations:** Cancer Research UK and Medical Research Council Oxford Institute for Radiation Oncology, Department of Oncology, University of Oxford, United Kingdom

**Keywords:** Prospective, Gating, Respiration, Motion, Reacquisition, Mouse

## Abstract

**Purpose:**

Multi-slice scanning in the abdomen and thorax of small animals is compromised by the effects of respiration unless imaging and respiration are synchronised. To avoid the signal modulations that result from respiration motion and a variable TR, blocks of fully relaxed slices are typically acquired during inter-breath periods, at the cost of scan efficiency. This paper reports a conceptually simple yet effective prospective gating acquisition mode for multi-slice scanning in free breathing small animals at any fixed TR of choice with reduced sensitivity to respiratory motion.

**Methods:**

Multi-slice scan modes have been implemented in which each slice has its own specific projection or phase encode loop index counter. When a breath is registered RF pulses continue to be applied but data are not acquired, and the corresponding counters remain fixed so that the data are acquired one TR later, providing it coincides with an inter-breath period. The approach is refined to reacquire the slice data that are acquired immediately before each breath is detected. Only the data with reduced motion artefact are used in image reconstruction. The efficacy of the method is demonstrated in the RARE scan mode which is well known to be particularly useful for tumour visualization.

**Results:**

Validation in mice with RARE demonstrates improved stability with respect to ungated scanning where signal averaging is often used to reduce artefacts. SNR enhancement maps demonstrate the improved efficiency of the proposed method that is equivalent to at least a 2.5 fold reduction in scan time with respect to ungated signal averaging. A steady-state magnetisation transfer contrast prepared gradient echo implementation is observed to highlight tumour structure. Supplementary simulations demonstrate that only small variations in respiration rate are required to enable efficient sampling with the proposed method.

**Conclusions:**

The proposed prospective gating acquisition scheme enables efficient multi-slice scanning in small animals at the optimum TR with reduced sensitivity to respiratory motion. The method is compatible with a wide range of complementary methods including non-Cartesian scan modes, partially parallel imaging, and compressed sensing. In particular, the proposed scheme reduces the need for continual close monitoring to effect operator intervention in response to respiratory rate changes, which is both difficult to maintain and precludes high throughput.

## 1 Introduction

MRI scanning in the abdomen and thorax of small animals is compromised by the effects of respiration motion. An isoflurane anaesthetized (1–3% in air and/or O_2_) normal healthy mouse takes snatched breaths of about 200 ms duration with a significantly depressed and often variable respiration rate, typically 40–80 breaths/min depending on the depth and duration of anaesthesia [[1], [2], [3], [4], [5]]. Prospective gating methods incorporating the automatic reacquisition of respiratory motion corrupted data have enabled highly efficient motion desensitised 3D scanning at short and constant TR in the mouse [6]. The methods adaptively track spontaneous changes in the respiration rate to maximise acquisition during all inter-breath intervals when respiration motion is minimal, and have been shown work very well in spoiled gradient echo and balanced SSFP scan modes. These methods, however, are not able to replicate the same level of *T*_2_ contrast offered by the multi-slice RARE scan mode, which is well known to be particularly useful for tumour visualization. This paper reports a conceptually simple yet effective prospective gating acquisition scheme for efficient multi-slice scanning in free breathing small animals at any fixed TR of choice with reduced sensitivity to respiratory motion.

For a spin echo scan with a 90° excitation the maximum SNR per unit time is achieved at the familiar TR = 1.26*T*_1_ [7] which corresponds to a 44% improvement over scanning with TR = 5*T*_1_. Respiration motion artefacts have previously been minimised in triggered multi-slice scanning through the acquisition of a fixed number of slice data during inter-breath periods which forces TR to be determined by the time taken to complete an integer number of breath cycles [[8], [9], [10], [11]]. A long TR is required to avoid the signal amplitude modulation due to variable *T*_1_ weighting that results from a short and variable TR, and which manifests as image ghosting. The scan efficiency is compromised since TR is long. Furthermore, unpredictable events such as periodic double breaths result in unavoidable corruption of data. In instances where a respiratory triggered approach is considered too inefficient, motion artefacts have been reduced with signal averaging [12]. A variable TR also confounds the quantification of metrics derived from magnetisation preparation schemes such as MT and CEST. Even a simple MT ratio measurement requires a fixed TR to be meaningful and reproducible [13].

Slice selective retrospective gating strategies in small animals typically acquire data with short TR such that each line of k-space data can be sampled repeatedly over several cardiac cycles for cardiac R-wave alignment. We are aware of only one report that uses retrospective gating in conjunction with scanning at TR > 80 ms to align data with inter breath periods in order to minimise the effect of respiration motion [14]. Scanning the mouse abdomen at TR 400 ms with a retrospective approach required 9 full repetitions to obtain a fully sampled data set from the 20 repetitions that were initially acquired in the absence of hindsight.

Alternative acquisition strategies such as radial, spiral and, in the case of the RARE scan mode, PROPELLER, are able to reduce the effect of physiological motions by oversampling the centre of k-space in a manner that essentially performs low spatial frequency signal averaging, albeit at the expense of scan time [[15], [16], [17]]. The reduced sensitivity of PROPELLER to respiration motion has enabled the ADC of mouse liver to be determined with an approximately three-fold reduction in standard deviation when compared to both ungated and respiration triggered RARE scan modes [17]. Although a good degree of motion correction is possible, the destructive interference between echoes of the RARE CPMG echo train that is caused by a loss of phase coherence cannot be recovered. In general, the effect of motion on pure frequency encoding methods is to spread the artefact energy in all directions, which is considered to be less detrimental to image quality than the phase encode ghosting exhibited by conventional Cartesian spin-warp methods. The prospective gating multi-slice method presented here is compatible with all of these acquisition schemes, and follows the guiding principle that the best way to remove artefacts is to avoid them altogether [18].

## 2 Methods

### 2.1 Multi-slice respiration gating at the optimum TR

Conventional multi-slice MRI is characterised by having a slice index counter that cycles more quickly than the projection or phase encode index counter. By assigning each slice its own specific projection or phase encode loop index counter it is possible to scan the slices continually at the optimum TR. When a breath is registered RF pulses continue to be applied but data are not acquired, and the corresponding counters remain fixed so that the data are acquired one TR later, providing it coincides with an inter-breath period. Each slice advances through its own projection/encoding loop whenever suitable data are acquired during an inter-breath period, as determined by the spontaneous respiration rate, and the scan only completes when all of the slices have acquired the specified number of projections/encodings.

**Fig. 1:**
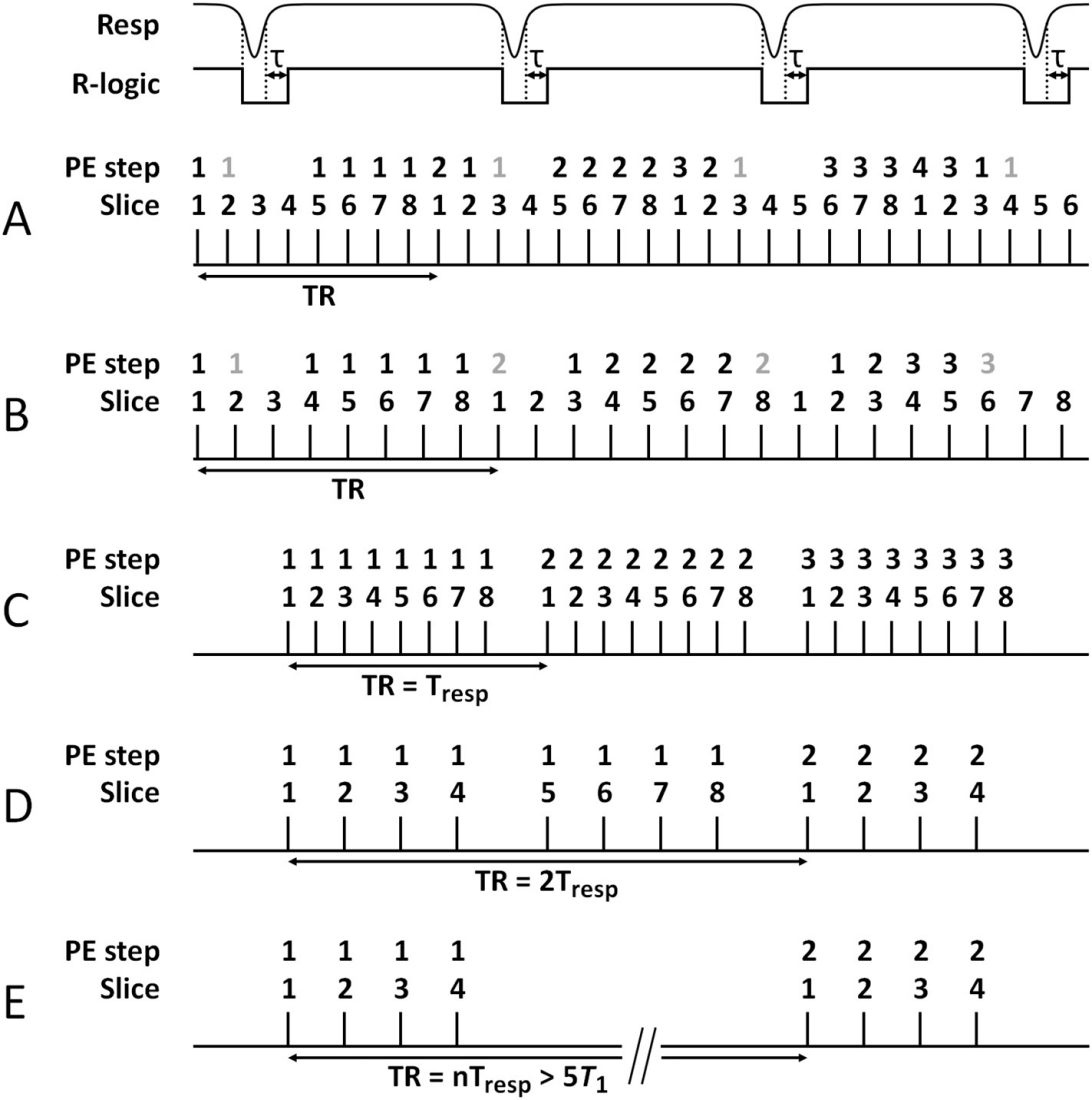
Diagrammatic representation of respiration gated scan modes for 8 example slices. A threshold is set on the analogue respiration signal (Resp) to generate the R-logic control signal. A user-variable post breath delay (τ) is used to ensure that motion artefact is minimised from the trailing portion of each breath. A, B: SPLICER scan modes set for two example TRs. The R-logic control signal is evaluated in real time. If the signal registers a breath, RF continues to be applied but data are not acquired, as indicated by the absence of a projection/phase encoding (PE) step. Each breath is already in progress at the time of detection using threshold breath detection. In the diagram a single respiration corrupted PE step (marked in grey) is reacquired the next time the slice falls favourably within an inter-breath period. C, D, E: Conventional modes where TR is equal to a single instantaneous breath interval (C), the sum of two successive instantaneous breath intervals (D), and the sum of n successive instantaneous breath intervals selected to allow full relaxation (E). In the conventional modes pulse sequences are suspended following acquisition of a predefined block of slices until triggered by a low to high switch of the R-logic signal.

Each breath is already in progress at the time of detection using threshold breath detection. The approach is refined to reacquire the slice data acquired during a user-defined time period before each breath is detected as this has been shown to dramatically improve image stability [6]. For convenience the acquisition scheme is referred to as SPLICER (Slice Projection Loop Index Counter Enabled Reacquisition) [19], and only the data with reduced sensitivity to respiratory motion are spliced together for image reconstruction. A diagrammatic representation of the SPLICER scan mode is shown for two different TRs in Fig. 1A and B and the scan modes that have previously been used to prospectively synchronise data acquisition with inter-breath periods are shown in Fig. 1C, D and E.

It has previously been reported that only small variations in physiological cycles result in sufficient asynchrony such that motion insensitive data can be sampled efficiently [20]. This is demonstrated by simulation for a normal distribution in Supplementary material. In practice, the respiration rate typically drifts and scans complete according to the time that is available for respiration insensitive scanning. Spreading the standard ungated scan time over the inter-breath periods that are available for respiration insensitive scanning defines an extended scan time. Analysis of real respiration traces suggests that at least 97% of SPLICER scans will complete within a 10% overhead of this extended scan time.

The prospectively gated 3D methods described previously operate at very short TR (<3 ms), and blocks of data with centre-out phase encoding are acquired to align the centre of k-space with inter-breath cardiac R-waves [6]. Only the two standard projection loop counters, one for each projection encoding direction, are required for efficient 3D sequence control including the automatic reacquisition of respiration corrupted data, in contrast to the requirements of the 2D multi-slice method presented here for which each slice has its own independent projection counter. In general, the multi-slice method is deployed at much longer TR which provides ample time for respiration logic signal evaluation before RF is applied to each slice. The overhead associated with real-time decision making is therefore more of an issue for the 3D methods.

### 2.2 In vivo

All animal studies were performed in accordance with the UK Animals (Scientific Procedures) Act of 1986 under licences approved by the UK Home Office and with the approval of the University of Oxford ethical review committee. Animals were housed in environmentally enriched (activity wheel or crawl ball, nesting material and chew sticks) IVC cages in groups of 5 per cage, in a 12 h day-night cycle facility maintained at 22 °C in 50% humidity. All mice had ad libitum access to certified food and tap water. Female, 8-week old CBA (n = 4) and C57BL/6 (n = 1) mice (Charles River) were used.

Anaesthesia was induced and maintained using isoflurane (1–4%) in room air supplemented with oxygen (80%/20% v/v) for MRI. Rectal temperature was monitored and maintained at 36 °C with an optical system (ACS-P4-N-62SC and OTP-M, Opsens Inc., Quebec, Canada) that provided feedback to a twisted pair resistive heating system developed for MR compatible homeothermic maintenance [21]. Respiration was monitored and maintained at 40–60 breaths/min using a pneumatic balloon (VX010, Viomedex Ltd, UK) positioned against the animal's chest and coupled to a pressure transducer. The respiration signal was passed to a custom-built gating device to generate a threshold based respiration gating control signal with additional duration of about 150 ms set to last until after the completion of each breath to reduce the sensitivity to respiratory motion.

A pancreatic tumour was derived from orthotopic injected KPC cells in a C57BL/6 mouse [22], and CaNT tumours were subcutaneously implanted [23] in 3 CBA mice, as part of separate studies.

### 2.3 MRI

MRI was performed on a 4.7 T 310 mm horizontal bore Biospec AVANCE III HD preclinical imaging system equipped with 114 mm bore gradient insert (Bruker BioSpin GmbH, Germany) or a 7.0 T 210 mm horizontal bore VNMRS preclinical imaging system equipped with 120 mm bore gradient insert (Varian Inc., CA). RF transmission and reception was performed with a 45 mm long 32 mm ID quadrature birdcage coil (Rapid Biomedical GmbH, Germany). The SPLICER scheme was implemented in RARE and magnetisation transfer prepared gradient echo scan modes with evaluation of the respiration gating signal preceding the acquisition or dummy scanning of each slice.

A user defined period of 150 ms determined the amount of slice data acquired immediately preceding the detection of each breath that was set to be reacquired. Combining the reacquisition with extension of the respiration gating control signal beyond each snatched breath results in a period of about 500 ms duration about each breath that is not available for data acquisition.

The scanner architecture of both systems necessitates that the exact amount of data to be acquired is specified at the start of a scan. Traditionally, for a full 2D multi-slice data set, this corresponds to the acquisition of *np* complex data points for each projection multiplied by *ns* slices multiplied by *nproj* projections. Increasing the projection loop size by a factor of four easily ensures that scans complete naturally with a full set of acceptable data and do not terminate prematurely.

To determine exactly which data should be used in image reconstruction each acquired data trace was timestamped (Bruker) or the projection counter index of each slice was passed to the parallel port of the spectrometer host computer for storage (Varian).

The stability of the SPLICER acquisition mode was tested (Bruker) with 10 repeats of a 2D multi-slice RARE scan with ETL 8, effective TE 24 ms, TR 2000 ms, THK 0.5 mm, 24 contiguous slices, FOV 32 × 32 mm^2^ and matrix 128 × 128. For comparison, 20 repeats of an ungated scan acquired in a virtually identical scan time were acquired with otherwise identical parameters. For tumour visualization 3 repeats of the SPLICER acquisition mode were acquired with fat saturation and effective TE 48 ms.

For gradient echo scanning with θ < 45°, the optimum TR is <0.33*T*_1_. Compatibility of SPLICER with steady-state magnetisation preparation was tested (Varian) in tumour bearing mice with a multi-slice gradient echo scan with TE 3.5 ms, TR 400 ms, FA 30°, THK 0.422 mm, 48 contiguous slices, FOV 27 × 27 mm^2^ and matrix 128 × 128. MTC preparation was performed with a 2 ms sinc 90° RF pulse applied downfield at +5 kHz from water followed by a 1 ms 100 mT/m crusher gradient.

The SPLICER acquisition schemes and reconstructions for both Bruker and Varian platforms are open source and freely available for download courtesy of the Bodleian Digital Library Systems and Services of the University of Oxford at https://doi.org/10.5287/bodleian:pvRrYE7Kk.

## 3 Results and discussion

**Fig. 2:**
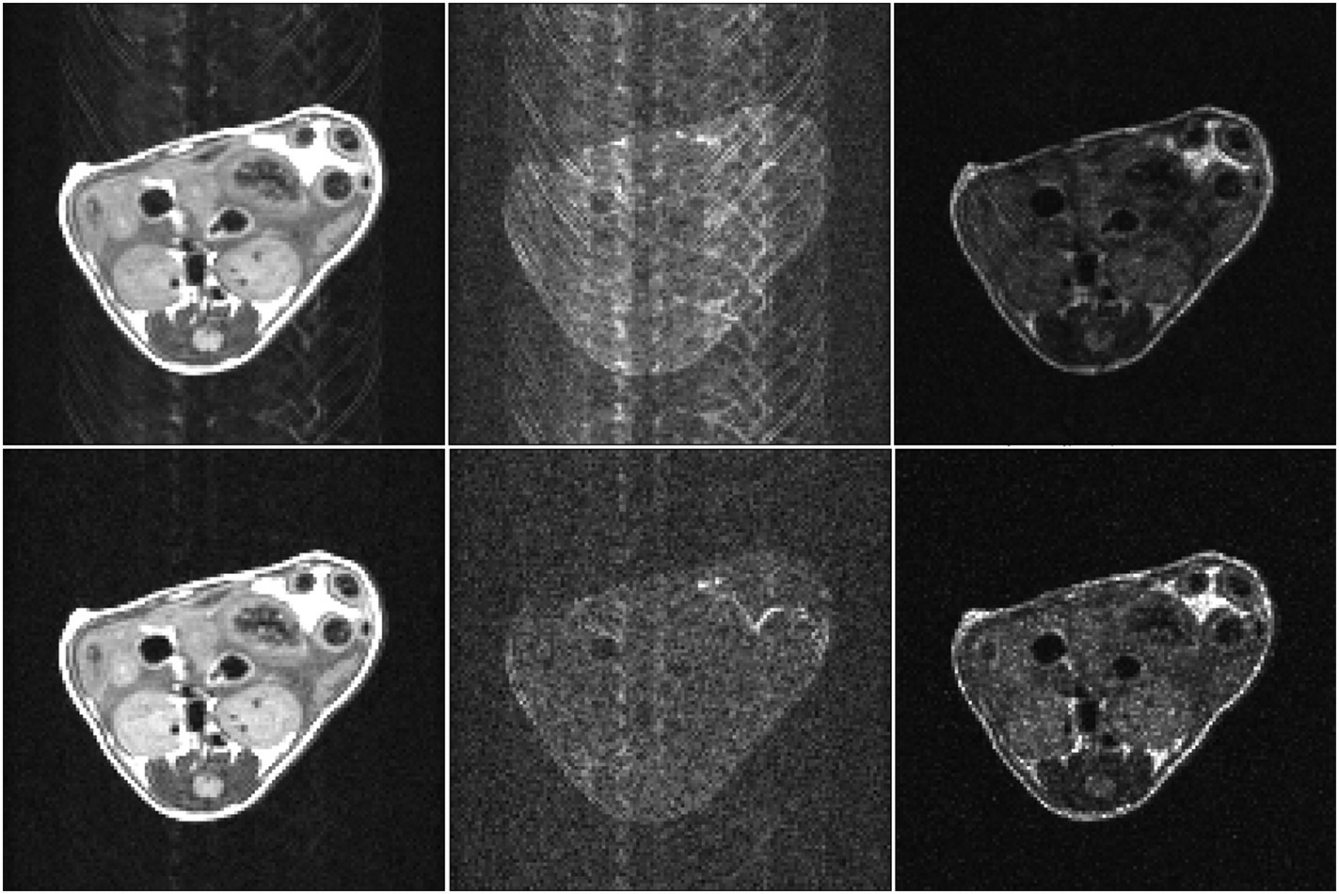
Mean (left column), standard deviation (middle column) and SNR (right column) maps from slice 15 through the kidneys of a mouse. The data are calculated from repeats of a RARE scan acquired with TR 2000 ms: 20 repeats of an ungated scan acquired in 10.8 min (top row); and 10 repeats of a gated scan using SPLICER acquired in 10.5 min (bottom row). The data of each column are displayed with the same intensity scaling.

Fig. 2 shows (from left to right) mean, standard deviation (SD) and SNR (mean/SD) maps calculated for an example slice through the kidneys of a mouse from repeats of a RARE scan acquired with TR 2000 ms. The top row shows results from 20 repeats of an ungated scan, and the bottom row shows results from 10 repeats of a respiration gated scan acquired in a virtually identical scan time using SPLICER. The SNR of each pixel was calculated according to the mean/SD obtained from a standard statistical analysis of the magnitude time course data for each pixel. This measure of SNR includes the effect of variance due to physiological movement as well as the generic system noise and eliminates the requirement to select a specific ROI for noise analysis. It is evident that the SPLICER scan results in considerably less motion induced ghosting and the residual artefacts are primarily due to gut motion and cardiac pulsation.

**Fig. 3:**
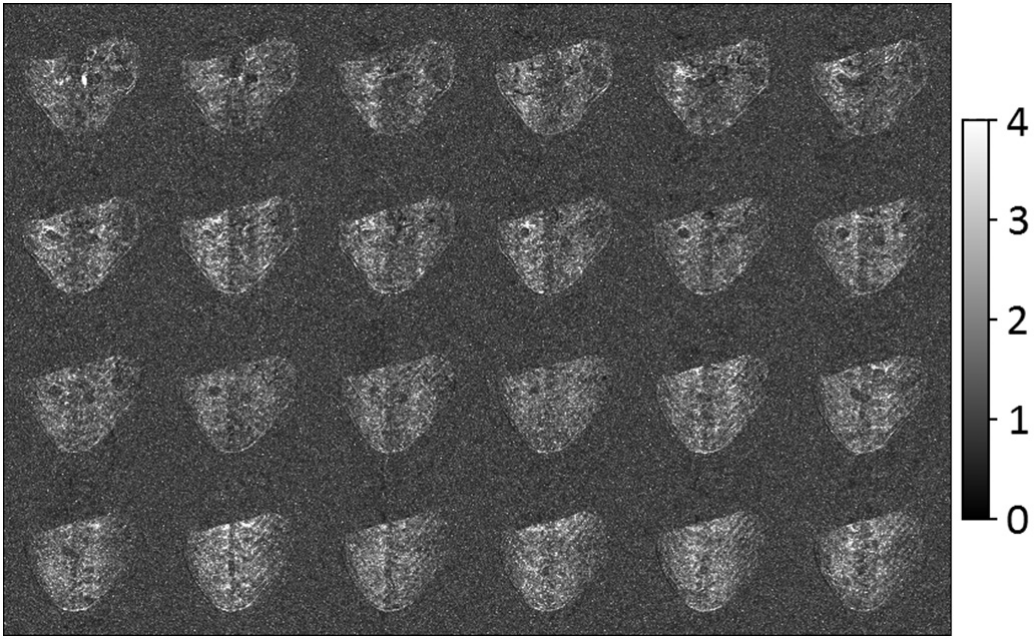
SNR enhancement maps for the SPLICER acquisition mode with respect to ungated scanning (SNR of SPLICER/SNR ungated scanning) for all 24 slices. The SNR of SPLICER is calculated from 10 repetitions and the SNR of ungated scanning is calculated from 20 repetitions which are acquired in essentially the same scan time.

Fig. 3 shows SNR enhancement maps for the SPLICER acquisition mode with respect to ungated scanning (SNR of SPLICER/SNR ungated scanning) for all 24 slices. The general ghosting displayed in the enhancement maps is a direct result of the respiration induced phase encode ghosting that is evident in the ungated SNR maps. The vertical bands of hypointensity are primarily a result of pulsatile blood flow in the major blood vessels that run through the imaging plane. The instabilities caused by pulsatile blood flow cannot be reduced by the SPLICER acquisition mode. The mean SNR enhancement across all 24 slices for a large (128 mm^2^ = 2048 pixels) circular ROI that fills a good portion of the body region in each slice was calculated to be 1.6. Considering that the ROI unavoidably includes some signal voids which register as unity, one has to conclude that, in this instance, ungated scanning would have to be performed for at least 2.5 times as long in order to achieve the same SNR as exhibited by the SPLICER acquisition mode. The observed SNR improvement will inevitably depend upon a number of factors including type of contrast encoding, extent of body motion, slice thickness, etc., but it is generally delivered because conventional signal averaging cannot recover motion-derived losses in SNR.

The assessment of temporal stability, rather than single frame examination, provides an unequivocal demonstration of the signal intensity stabilisation and increased image fidelity of the proposed method. The data sets are publically available at https://doi.org/10.5287/bodleian:pvRrYE7Kk in NIfTI-1.1 format (http://nifti.nimh.nih.gov/) and can be viewed with ImageJ (https://imagej.nih.gov/ij/). It is particularly instructive to inspect the stability of data by selecting a slice and scrolling through the time course. It can readily be seen that the single slice data presented in Fig. 2 are entirely representative.

**Fig. 4:**
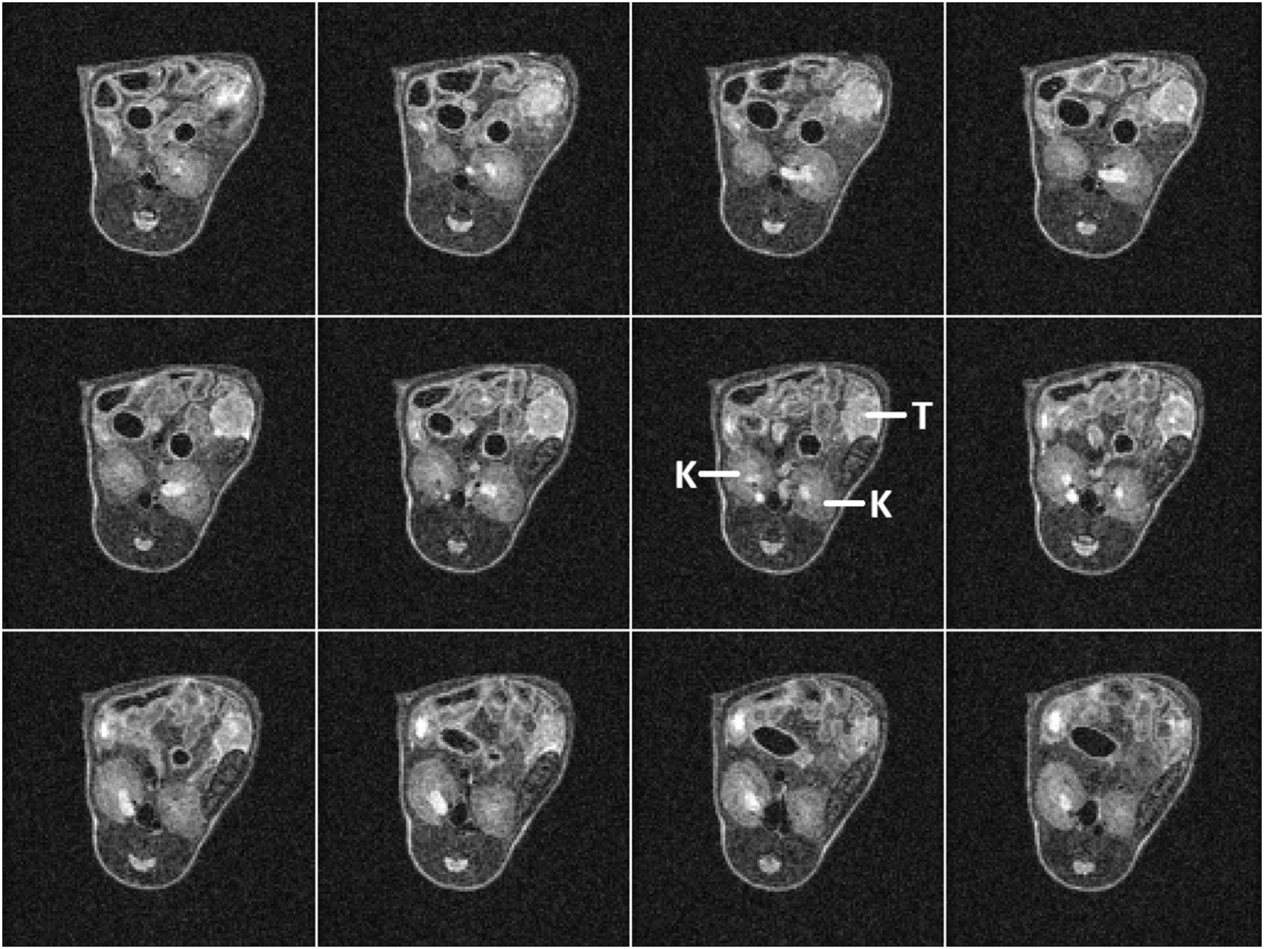
Contiguous slices in a pancreatic tumour (T) bearing mouse showing the mean of 3 repeats of a SPLICER RARE scan acquired with TR 2000 ms, effective TE 48 ms and fat saturation in a total imaging time of 2.5 min. The kidneys are marked K.

Fig. 4 shows 12 contiguous slices from an orthotopic pancreatic tumour bearing mouse from 3 repeats of SPLICER RARE scan acquired in 2.5 min. The data of all slices are devoid of motion artefact and exhibit good tumour delineation which can be used to plan MR guided radiotherapy treatment [24,25]. In this application it is particularly crucial to minimise scan time to ensure that long term animal motions such as peristalsis, bladder filling and body droop do not lead to significant deformation of the body in between the start of imaging and the delivery of radiotherapy. It is also particularly important to minimise image artefacts since they can compromise image registration and result in misdirected treatments.

For quantification of MRI metrics in general it is important to minimise scan time as precision is improved when longer term physiological instabilities are minimised. We have previously observed, albeit in a 3D scan mode, that measurement of *T*_1_ in a number of organs have improved precision when acquiring data in a shorter scan time whilst minimising the effects of respiration motion [26]. For 2D multi-slice scanning the SPLICER acquisition mode is the most efficient way to minimise the effects of respiration motion for quantitation.

It must be noted, however, that through slice motion during the breath provides an opportunity for inadvertent excitation of the wrong slices. It is not straightforward to formally assess the extent to which this will confound quantitation as it will depend on many factors including slice position, slice profile, slice thickness, and depth of anaesthesia. For parts of the body where significant through slice axis motion does occur, e.g. the thorax and liver, it is possible to suspend slice selective RF excitation during the breath whilst maintaining delivery of magnetisation preparation pulses as necessary [27,28]. This requires scans to be operated in a near fully relaxed mode which results in long scan times, but the real-time and adaptive tracking of the respiratory interval by the proposed method ensures that the scan efficiency is always maintained. Nevertheless, if possible, we would always choose to avoid the complications of through slice motion by deploying a short TR 3D method.

**Fig. 5:**
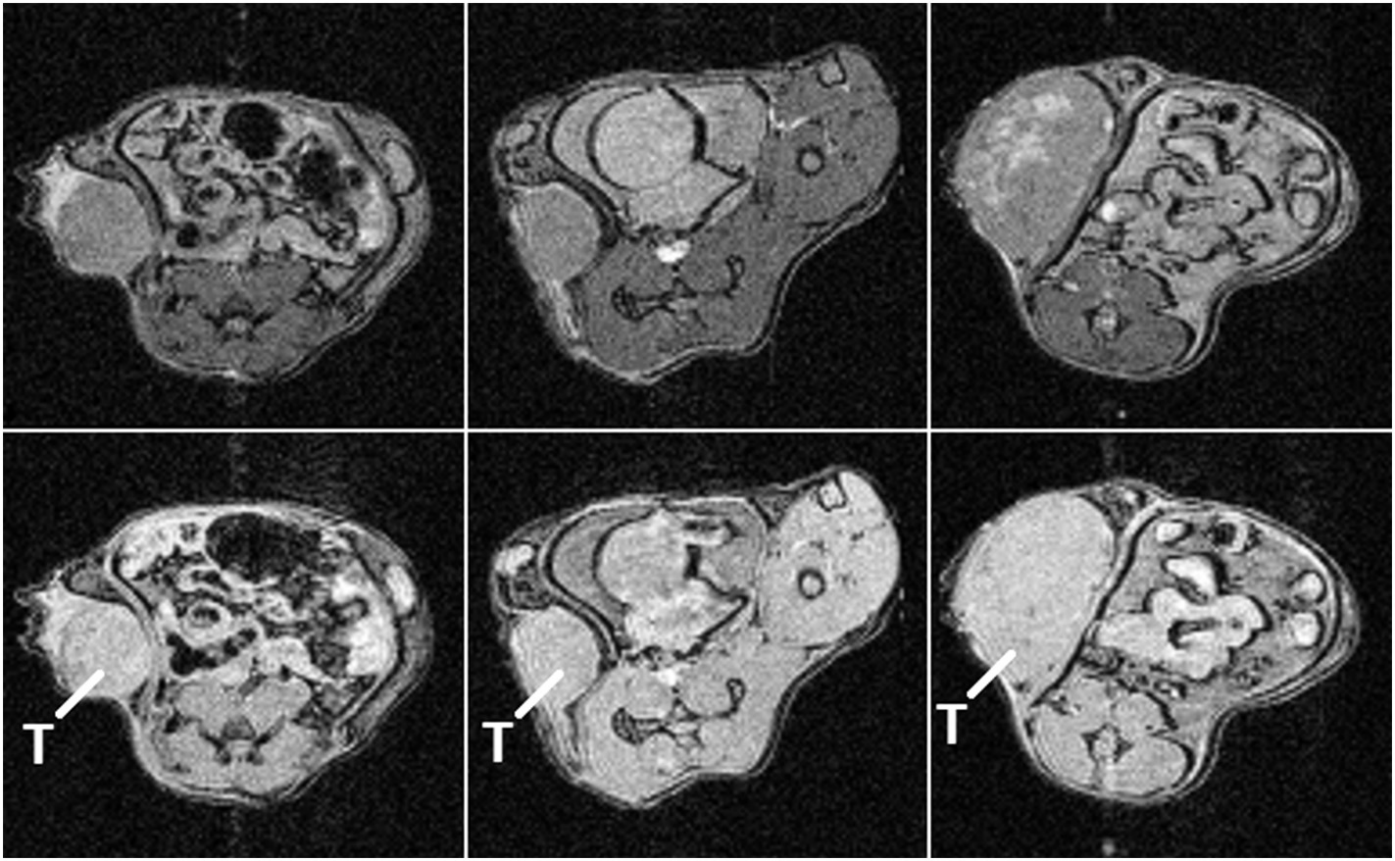
A single slice from SPLICER gradient echo scans in 3 CaNT tumour (T) bearing mice with (top row) and without (bottom row) steady-state MTC preparation. The data are all displayed with the same intensity scaling.

The proposed method naturally enables global steady-state magnetisation preparation schemes that are applied in the absence of slice selection, such as MTC and CEST, to be maintained through periods of bulk motion. Fig. 5 shows that good image quality is produced when using gradient echo SPLICER in conjunction with a steady-state MTC preparation scheme which shows darkening of the muscle and CaNT tumours relative to images acquired without MTC preparation. The CaNT tumour in the third column shows MTC delineating regions where it is suspected that necrosis has occurred and fluid has filled the resulting cavities. Other preparation schemes are, of course, more forgiving and do not need to be applied with such fixed regularity. For example, fat saturation only requires the fat magnetisation to be saturated immediately prior to data acquisition.

## 4 Conclusion

The proposed prospective gating acquisition scheme enables efficient multi-slice scanning in small animals at the optimum TR with reduced sensitivity to respiratory motion. The method has been implemented and demonstrated in multi-echo and magnetisation prepared spin-warp scans, and is compatible with a wide range of complementary methods including non-Cartesian scan modes and reduced data acquisition techniques. In particular, the proposed scheme reduces the need for continual close monitoring to effect operator intervention in response to respiratory rate changes, which is both difficult to maintain and precludes high throughput.

The following is the supplementary data related to this article.

**Supplementary Fig. 1:**
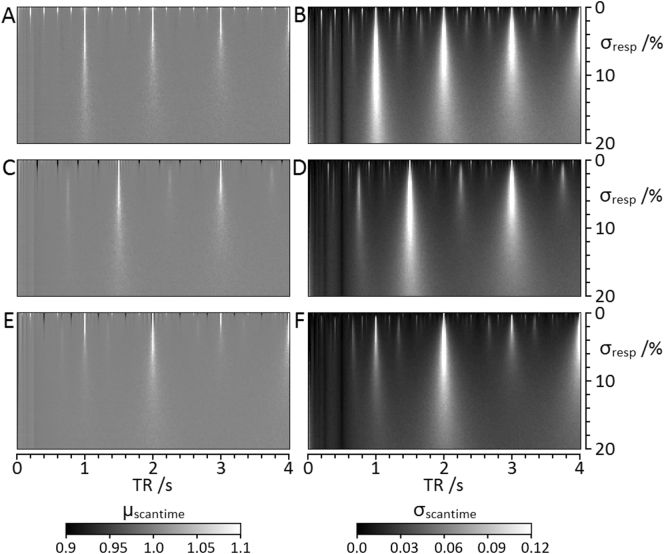
Simulations of SPLICER acquisitions showing mean and standard deviation scan times for 1000 repeats of the acquisition of 128 respiration insensitive projections. **A**,**C**,**E**: mean scan time (μ_scantime_). **B**,**D**,**F**: standard deviation of scan time (σ_scantime_). Scan cycles were simulated in the presence of respiration patterns characterised by breath motion duration 0.5 s and mean respiration interval (μ_resp_) of 1.0 s (**A**,**B**), 1.5 s (**C**,**D**) and 2.0 s (**E**,**F**) with normal distribution about the mean. The scale bars are displayed in multiples of the mean scan time available for respiration insensitive scanning corresponding to 2×, 1.5× and 1.33× the conventional ungated scan times for μ_resp_ = 1.0 s, 1.5 s, and 2.0 s respectively. Simulations were performed with standard deviation of the respiration intervals (σ_resp_) ranging from to 0.2% to 20% of μresp in steps of 0.2%, and for TR ranging from 0.02 s to 4.0 s in steps of 0.02 s. Each simulation was initiated exactly coincident with the centre of a breath which is considered to be the most favourable time within the respiration cycle for maintaining synchrony between the respiration and scan cycles and, therefore, invoking reacquisition and prolonging the scan time.
